# Scanning micro-resonator direct-comb absolute spectroscopy

**DOI:** 10.1038/srep35541

**Published:** 2016-10-18

**Authors:** Alessio Gambetta, Marco Cassinerio, Davide Gatti, Paolo Laporta, Gianluca Galzerano

**Affiliations:** 1 Dipartimento di Fisica–Politecnico di Milano, Piazza Leonardo da Vinci 32, 20133 Milano, Italy; 2 Istituto di Fotonica e Nanotecnologie–CNR, Piazza Leonardo da Vinci 32, 20133 Milano, Italy; 3 IMRA America Inc., 1044 Woodridge Avenue, Ann Arbor, Michigan 48105, USA

## Abstract

Direct optical Frequency Comb Spectroscopy (DFCS) is proving to be a fundamental tool in many areas of science and technology thanks to its unique performance in terms of ultra-broadband, high-speed detection and frequency accuracy, allowing for high-fidelity mapping of atomic and molecular energy structure. Here we present a novel DFCS approach based on a scanning Fabry-Pérot micro-cavity resonator (SMART) providing a simple, compact and accurate method to resolve the mode structure of an optical frequency comb. The SMART approach, while drastically reducing system complexity, allows for a straightforward absolute calibration of the optical-frequency axis with an ultimate resolution limited by the micro-resonator resonance linewidth and can be used in any spectral region from UV to THz. We present an application to high-precision spectroscopy of acetylene at 1.54 *μ*m, demonstrating performances comparable or even better than current state-of-the-art DFCS systems in terms of sensitivity, optical bandwidth and frequency-resolution.

Over the past decade high-resolution and broadband spectroscopy has received a major boost from the advent of optical frequency combs (OFCs), highly-coherent light sources constituted by an array of evenly spaced optical narrow lines whose absolute frequencies are known with a fractional accuracy of 10^−15^ or even better^1^,^2^. Direct frequency comb spectroscopy (DFCS) exploits high-speed (or parallel) approaches in order to simultaneously detect such a massive set of extremely accurate channels, allowing for ultra-broadband and high speed spectroscopic investigations^3^,^4^,^5^. Since the first DFCS experiment^6^, several detection methods have been developed in order to increase the frequency resolution without sacrificing the broadband requirements^7^,^8^,^9^,^10^,^11^,^12^,^13^,^14^,^15^,^16^,^17^,^18^,^19^. Only three DFCS techniques have however demonstrated a frequency resolution down to the comb-tooth level. The first method is the dual-comb or coherent multi-heterodyne spectroscopy, employing two OFC sources with slightly detuned comb-line spacing^7^,^20^ that produce an interferogram in the time domain. Spectroscopic informations on the absorption features of the sample are retrieved by Fourier-transform of the interferogram. While providing real-time acquisition capabilities, this technique relies on sophisticated frequency stabilization servo loops or adaptive real time sampling to overcome the frequency noise and relative drifts between the two OFCs^21^,^22^,^23^,^24^. State-of-the-art systems in the near infrared region have shown to resolve the single comb tooth with a frequency resolution of 200 kHz over optical bandwidths as broad as 10 THz^23^. Very recently, a compact dual-comb system based on intensity modulated CW laser has demonstrated an even better resolution of 13 kHz over an optical bandwidth of 0.3 THz^25^. A second approach is based on the combination of two orthogonal spectral-dispersing elements projecting the resolved modes of a single comb onto a bi-dimensional optical sensor such a CCD. By exploiting a virtually imaged phased array (VIPA)^26^ as a highly dispersing element, several implementations have been proposed able to detect single comb lines with a frequency resolution in the range 0.6 to 1 GHz^10^,^27^,^28^,^29^. A variant of the approach is based on the substitution of the VIPA with a Fabry-Pérot (FP) resonator^11^,^12^,^30^,^31^. The resonator modes are scanned across the frequency comb like a Vernier in frequency space and groups of comb lines containing the spectroscopic information are streaked on a two-dimensional detector array through the use of a scanning mirror synchronized to the cavity sweep cycle. In principle this technique allows for extremely high resolutions, down to the Hz level, however several technical shortcomings mainly ascribable to the finite resolution of the CCD camera limit its spectral resolution to 1.1 GHz^32^. The use of a CCD camera also prevents this technique to be adopted in spectral regions where fast, high-resolution imaging devices are not available (XUV and THz), while the unavoidable aliasing due to the Vernier effect needs further experimental arrangements to achieve the absolute calibration of the optical-frequency axis^29^. Finally, the cavity length needs to be matched to the optical frequency comb-mode spacing in order to achieve the desired Vernier ratio. This makes the design of the detection stage tightly connected to the characteristics of the laser source employed, feature that may be undesirable in remote sensing applications. As a third technique, Fourier-Transform Spectroscopy (FTS) coupled to OFC sources has become a powerful combination enabling the measurement of broadband spectra with comb-tooth level resolution down to 28 MHz^18^,^33^,^34^. As FTS is based on a Michelson interferometer with a scanning delay line on one arm, reaching high resolution requires very long delay lines of up to ~10 m, which implies measurement times of few minutes and an extended optical layout that is highly sensitive to vibrations. Recently, the resolution limit of FTS has been overcome by cutting the interferogram length in such a way that the measurement points in frequency domain overlap with position of the comb modes. This last approach allows spectroscopic measurements with acquisition time and interferometer length reduced by orders of magnitude and with frequency scale accuracy given by the comb^35^.

In this letter, we present a different DFCS approach based on a scanning FP micro-cavity resonator, SMART DFCS (Scanning Micro-cAvity ResonaTor DFCS), that is able to detect the spectrum of the OFC with a resolution much better than the comb-mode spacing, limited by the micro-resonator resonance linewidth. The proposed approach can be seen as a cavity Vernier spectroscopy with a Vernier ratio, the ratio between the cavity free-spectral range and the comb repetition rate, extremely large, which corresponds to the useful condition that only one comb mode resonates with the cavity^12^,^30^. The SMART DFCS significantly reduces the system complexity and allows direct implementation of this method to any spectral region from 20 *μ*m to 200 nm. A further spectral extension down to THz region will be feasible in the next years when high-finesse THz resonators will be available.

Figure 1 sketches the operating scheme. An OFC, whose output spectrum is constituted by evenly spaced optical lines at frequencies *ν*_*comb*_ = *f*_0_ + *nf*_*r*_, where *f*_0_ is the comb offset frequency, *f*_*r*_ is the comb line spacing, and *n* is the order of the comb line, is coupled to a FP micro-resonator with a free-spectral-range *FSR* = *c*/(2*d*) ≫ *f*_*r*_, where *d* is the mirrors separation and *c* is the speed of light. The FP micro-resonator finesse, 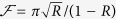 where *R* is the reflectivity of the cavity mirrors, is such that 
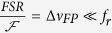
 corresponding to the case where only one comb mode is resonant with the cavity at a time. The FP transmission versus scanning time corresponds therefore to the classical optical spectrum analyzer configuration, which represents the spectral convolution between the FP Airy function and the comb spectrum. In order to avoid spectral aliasing between the adjacent transmission orders of the FP, the optical bandwidth of the OFC must be narrower than the cavity FSR, condition that may not be fulfilled for many typical OFCs even for a very short spacing of the cavity mirrors. For this reason an optical-tunable-filter (OTF), either before or after the FP micro-resonator, has to be used in order to select the appropriate spectral portion of the OFC to be detected.

As sketched in Fig. 1, environmental and electrical noise translates into a temporal jitter of the micro-cavity transmission modes. This effect is amplified by the very short cavity length, being the cavity detuning 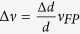, and prevents the frequency axis from being linearly mapped onto the acquisition time scale. A correction algorithm is therefore needed to compensate for cavity jitter and reconstruct the optical frequency axis. This is done in post-processing by taking advantage of the clearly resolved comb modes which act as a reference ruler for frequency remapping. For the purpose of absolute frequency calibration of the optical axis a free-running CW laser, with a frequency stability better than the comb spacing, is combined with the OFC. This CW source acts as a frequency marker to measure in real time the comb-mode order, given by the simple relation *N* = (*ν*_*CW*_ − *f*_0_)/*f*_*r*_. Exploiting the absolute frequency scale of the OFC, the position of all the measured comb teeth is known with the accuracy of the comb source even in the presence of micro-resonator dispersion, CW laser frequency fluctuations, and cavity length jitter during the scanning time. Therefore, effective averaging of the recorded spectra can be performed to boost the signal-to-noise ratio (SNR) of the detection system and to improve the sensitivity of the technique.

## Results and Discussion

**Figure 2:**
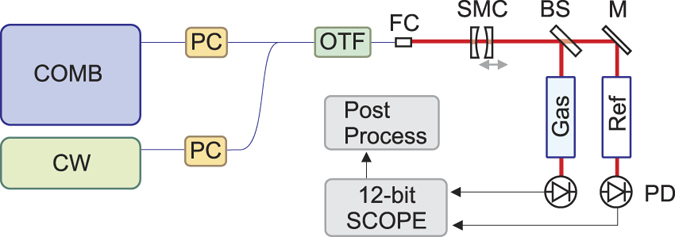
Experimental setup of the SMART DFCS method using an Er-fiber OFC at 1.55 *μ*m. CW: extended cavity laser diode. COMB: self-referenced optical frequency comb. PC: polarization control. OTF: optical tunable filter. FC: free space fiber coupler. SMC: scanning micro-cavity. BS: beam splitter. M: Mirror. Gas: C_2_H_2_ filled cell. Ref: empty reference cell. PD: fast InGaAs photo-detector. Cavity transmission vs time is acquired by a 12-bit digital oscilloscope and sent to a Matlab-equipped personal-computer for post processing.

Feasibility of the SMART DFCS has been demonstrated by high-precision spectroscopy of acetylene at around 1.55 *μ*m. The comb source adopted is a self-referenced Er:fiber laser frequency comb providing a 0.5 W output power, with a 250 MHz pulse repetition frequency. Both *f*_0_ and *f*_*r*_ are stabilized against a GPS-disciplined Rb Radio-Frequency standard. The measurement setup is sketched in Fig. 2. The comb is combined with the output of a tunable CW laser with ~60 MHz frequency accuracy, filtered by the OTF, coupled to a high finesse (~50,000) FP micro-cavity and, after passing through a Herriot-type multipass absorption cell (33-m interaction length) filled with C_2_H_2_, is shined onto a 2 MHz bandwidth InGaAs photo-detector. A reference arm constituted by an empty cell and an identical detector is used for calibration, normalization and background removal. A spacing of ~150 *μ*m between the cavity mirrors ensures a free-spectral-range limited bandwidth of the order of 1 THz or 8 nm at a central wavelength of 1.55 *μ*m. The resonance frequency sweep is performed by continuously scanning the cavity-mirrors distance through a periodic voltage ramp applied to a piezo-electric transducer. As mentioned before, the micro-cavity scan is free-running and completely independent of comb repetition frequency *f*_*r*_.

**Figure 3:**
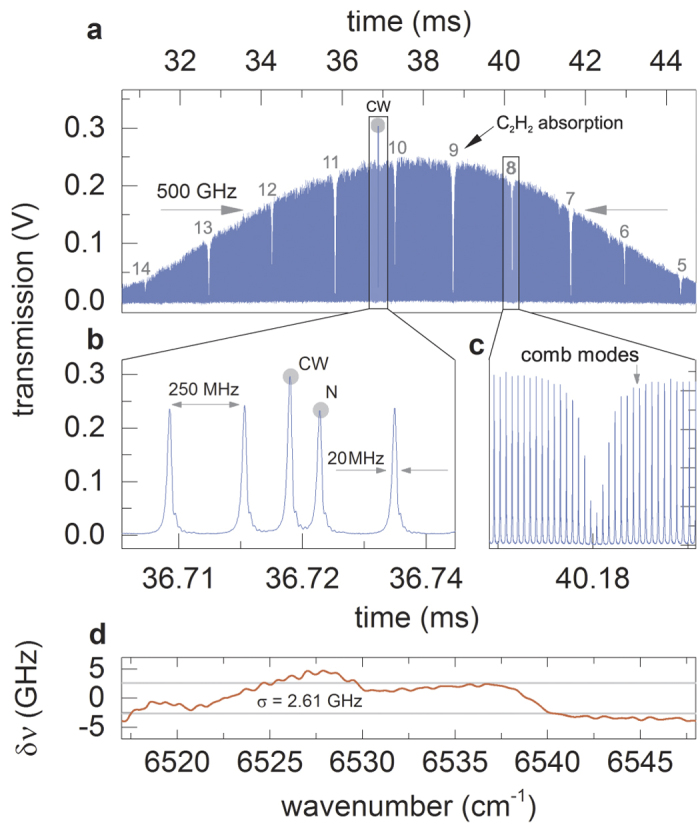
(**a**) Micro-resonator transmission versus scanning time. Both OFC and CW laser spectra are detected in the time-trace. A 10-cm long cell filled with C_2_H_2_ at 50 mbar is placed along the beam path. Several lines (numbered from 5 to 14) of the C_2_H_2_ P-branch absorption band are clearly visible as a modulation of the OFC spectrum. Middle panels: (**b**) temporal zoom around the CW laser showing the 20-MHz frequency resolution (FWHM) and the comb line spacing; (**c**) a detail of the resolved comb modes modulated by the P(8) rotovibrational absorption of the C_2_H_2_; (**d**) Micro-resonator jitter measured as the frequency deviation of the comb-peak position from a perfect linear dependence vs optical frequency.

Figure 3 shows a sample spectrum as acquired through a 12 bit oscilloscope by scanning the cavity length with a span Δ*d* of ~0.77 *μ*m in 14 ms (20-ms full scanning period), resulting in a scanning speed of 66 GHz/ms. A final resolution of ~20 MHz can be estimated for the adopted configuration as a result of mirror distance and cavity finesse. Individual comb modes as well as the CW laser line are clearly resolved, as shown in Fig. 3(b). Absorption features of the C_2_H_2_ gas are clearly visible as a modulation of the comb mode intensity (see Fig. 3(c)). A measure of cavity jitter and dispersion, as retrieved by the post-processing compensation and reconstruction algorithm, is reported in Fig. 3(d), showing the difference between the effective center frequency of each comb tooth and its value, as retrieved from the oscilloscope time scale without any correction. It is worth noting that without any environmental isolation of the microcavity, the measured frequency jitter shows a root-mean-square (r.m.s.) value of 2.6 GHz in an optical frequency scan of ~930 GHz, corresponding to a r.m.s. cavity length jitter of 2.2 nm.

**Figure 4:**
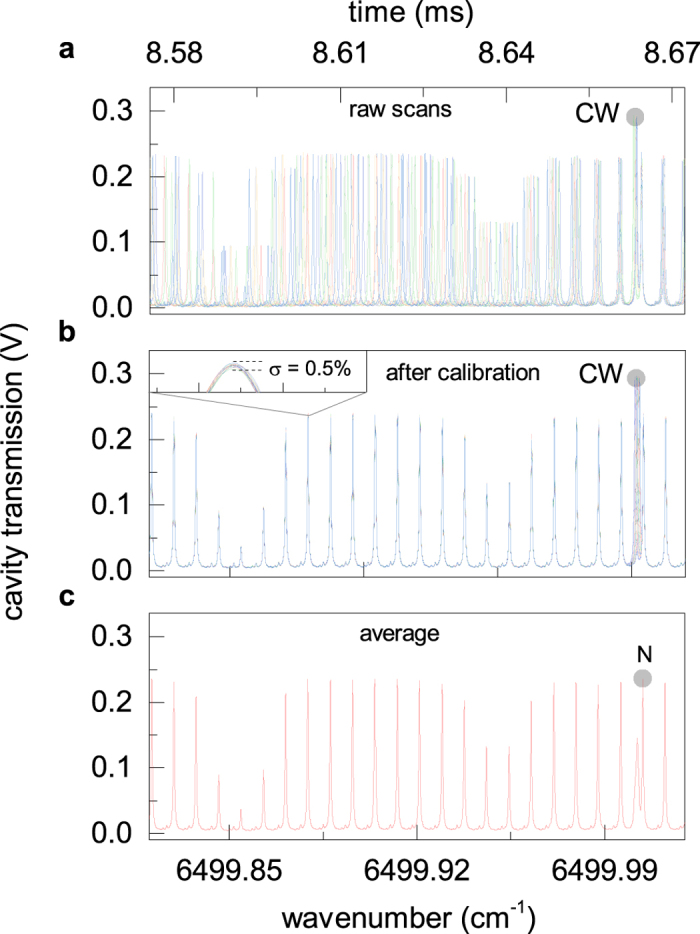
(**a**) 20 consecutive time-trace acquisitions as recorded by the oscilloscope. Each isolated peak corresponds to a single comb mode. The CW laser line can be distinguished as the highest peak. Two C_2_H_2_ absorption features are clearly visible as a modulation of the comb spectrum. (**b**) Measurements after jitter-compensation and absolute frequency calibration. Inset: RMS intensity noise of a comb peak. (**c**) Average of the 20 calibrated measurements.

**Figure 5:**
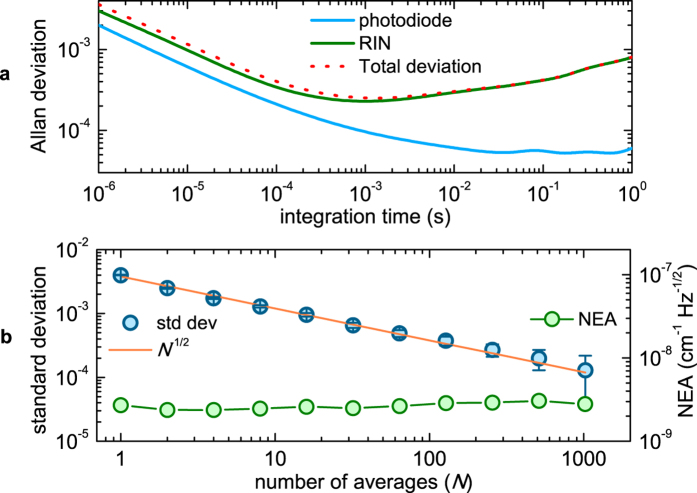
(**a**) Allan deviation (red dotted curve) versus integration times compared to that of the RIN of comb source (green curve) and of the noise background given by the photodetector (light blue curve). (**b**) Standard deviation (left axis) of the normalized transmission spectra versus the number of averages, *N*, as compared to the inverse square-root law expected for a normal noise distribution (orange curve). Corresponding NEA (right axis) as a function of the number of averages.

Figure 4(a) shows a spectral detail around the CW reference frequency of 20 raw consecutive acquisitions recorded with fixed values of comb offset and repetition frequencies ( *f*_0_ = 21 MHz and *f*_*r*_ =  250 MHz). The time base of each measurement has been translated in order to match the position of the *N*^*th*^-order comb tooth, next to the CW laser peak. Mainly due to the microcavity jitter, the recorded spectra are not overlapped in frequency and effective averaging can not be performed. Figure 4(b) reports the same 20 measurements after remapping and absolute frequency calibration. A perfect frequency overlapping of these spectra is obtained with a SNR of the single measurement limited by both the RIN of the comb and the photodetector noise at levels of about 0.4% and 0.3%, respectively, in a measurement bandwidth from 50 Hz to 2 MHz. The average of the 20 spectra is plotted in Fig. 4(c), showing also the comb mode *N* used for absolute calibration. In the averaging the CW laser line is smeared out and its peak value is decreased due the frequency noise along each sweep, whereas the comb spectrum shows an unchanged frequency resolution of 20 MHz. Figure 5(a) shows the Allan deviation of the normalized photodiode output as a function of the integration time compared to the contributions of the comb RIN and of the photodetector noise. For integration times shorter than 0.1 ms the Allan deviation is limited by the white noise contributions of both RIN and photodetector noise and it reaches a minimum level of 3·10^−4^ for an integration time of 1 ms. For longer integration times the Allan deviation is totally limited by the pseudo random-walk noise contribution of the comb RIN. To improve the SNR by keeping constant the 2-MHz measurement bandwidth (0.5 *μ*s integration time) it is necessary to average over many subsequent spectra. Figure 5(b) shows the standard deviation of the normalized transmission spectra as a function of the number of averages (*N*) acquired with a measurement bandwidth of 2 MHz. At *N* = 1 the deviation amounts to 4 · 10^−3^, in perfect agreement with the Allan plot over the corresponding time window; at increasing *N*, it decreases with the square root of *N* as expected for a normal noise distribution, attaining a value of 1.3 · 10^−4^ (SNR = 7700) at *N* = 1000, corresponding to a measurement time of 20 s. Figure 5(b) reports also the noise-equivalent averaged absorption coefficient per comb line, NEA, defined as 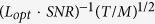, where *L*_*opt*_ (33 m) is the multipass-cell optical path, *SNR* is the signal-to-noise ratio, *T* is the measurement time (defined as *N* times the 20-ms microresonator full scanning period), and *M* (4000) is the number of resolved comb lines. The NEA at 1-s time-averaging per comb line is 2.7 · 10^−9 ^cm^−1^ Hz^−1/2^, where *SNR* = 1810.

**Figure 6:**
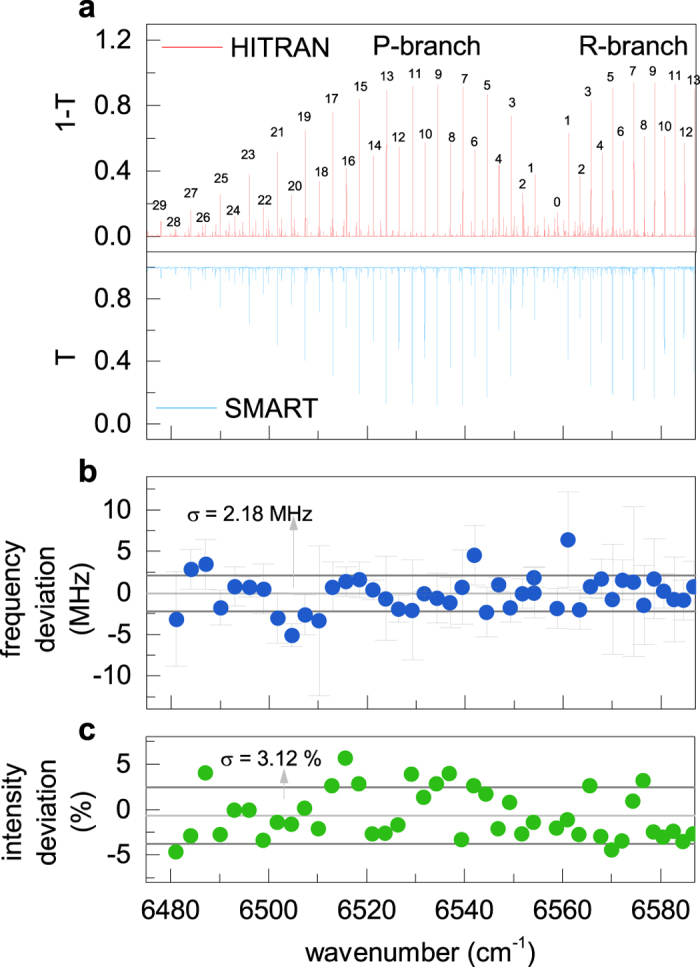
(**a**) Bottom panel: C_2_H_2_ spectrum at room temperature, *p* ≃ 0.045 mbar, optical path *L*_*opt*_ = 33 m, obtained via juxtaposition of ten spectrally adjacent SMART measurements. Top panel: HITRAN simulation. (**b**) frequency deviation of the retrieved line center frequency with respect to the HITRAN value showing a rms of ~2 MHz; (**c**) fractional deviation of the measured peak absorption with respect to the HITRAN simulation showing a rms of ~3%.

In order to overcome the 8-nm bandwidth limit of the single sweep, consecutive measurements have been performed with the same 1-THz micro-cavity FSR, tuning the center wavelength of the OTF to scan adjacent portions of the spectrum and to extend the overall optical bandwidth investigated. We chose a narrower ~2 nm filter bandwidth for each single-scan in order to keep an almost constant signal-to-noise ratio along the whole span. A C_2_H_2_ spectrum at a pressure of ~4.5 · 10^−2 ^mbar, obtained by combining 15 measurements performed in 2-nm broad adjacent spectral portions, is shown in Fig. 6(a) and compared to the spectrum simulated by HITRAN database^36^. Despite the low optical-frequency sampling, due to the 250 MHz comb spacing, very close to the ~450 MHz average linewidth of the absorption feature to be detected, the high SNR and the absolute frequency calibration of the SMART method allow for high-precision measurements of both line intensities and center frequencies. Figure 6(b,c) show, respectively, the deviation between the measured and computed line center frequency and the fractional deviation between the measured and simulated absorption peaks, showing rms values of 2 MHz and 3%, respectively, mainly limited by statistical contributions. Features as close as ~600 MHz such as the P1e and R10f lines (respectively at 6554.111497 and 6554.131 cm^−1^) have been detected easily with very good accuracy, with regards to both centerline position (respectively ~0.1 MHz and ~2.1 MHz) and transmission intensity (~1% and ~3%).

**Figure 7:**
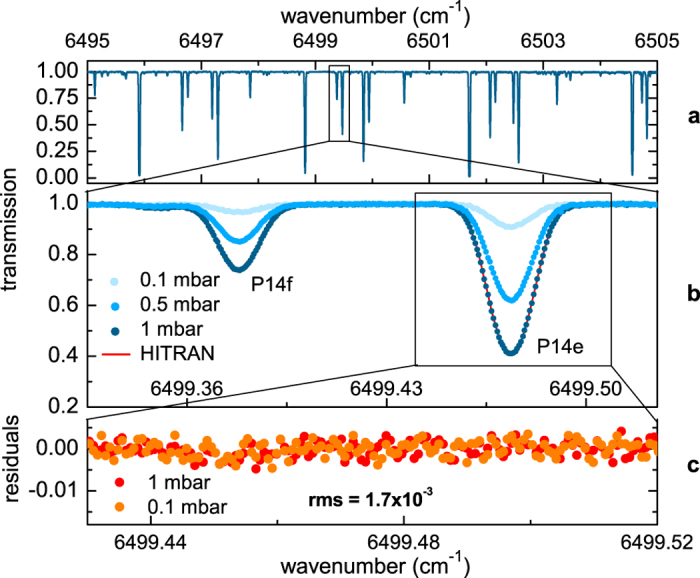
High resolution C_2_H_2_ SMART-DFCS spectrum (room temperature, *L*_*opt *_= 33 m) at three different pressures. Each spectrum is the result of twelve interleaved measurements, with frequency comb spectral detuning of 19.5 MHz between each measurement. (**a**) Full measurement spanning ~9 cm^−1^ (270 GHz); (**b**) particular around the P14f and P14e absorption lines over ~0.2 cm^−1^ span (dots). A simulation based on HITRAN databases is also shown (red line); (**c**) residuals from the HITRAN fit of the P14e line at 0.1 and 1 mbar.

To overcome the intrinsic sampling spacing limit set by the comb-teeth spacing, sequential interleaved measurements have also been performed by scanning the comb repetition rate. Figure 7(a) shows three C_2_H_2_ spectra recorded, respectively, at 0.1 mbar, 0.5 mbar, and 1 mbar. By choosing a frequency step of 19.5 MHz, corresponding to a Δ*f*_*rep*_ = 25 Hz, the whole 250 MHz spacing between adjacent comb modes has been covered by twelve consecutive measurements at each frep/frequency value. As shown in Fig. 7(b), the HITRAN simulation agrees very well with the experimental points. In particular, Fig. 7(c) shows the residuals from the HITRAN fit (using a conventional Voigt lineshape) of the P14e line (line intensity 2.3 · 10^−22 ^cm/molecule) at 0.1 and 1 mbar. No lineshape distortion can be appreciated in the residuals although the pressure is changed by one order of magnitude (peak absorption increases from 9% to 60%). Same results are obtained also for all the investigated lines although a w-shape distortion at around the line resonance frequency can be observed for line intensities higher than 10^−21 ^cm/molecule even at the lowest pressure of 0.1 mbar. This feature reveals the inappropriateness of the Voigt profile, i.e. the presence of more subtle narrowing effects on the experimental data, rather than an instrumental distortion. The high-precision and resolution performance of the SMART method can be further appreciated by the capability to measure the low pressure-shift coefficients of the investigated C_2_H_2_ lines: as a demonstration, an identical coefficient of −0.75(5) MHz/mbar has been measured for the first time for both P14f and P14e lines of Fig. 7(b), for pressure values up to 50 mbar. Control measurements have been performed on the P22 line, providing a pressure-shift coefficient of −0.388(18) MHz/mbar, in very good agreement with previous results^37^.

## Conclusion

By combining in an efficient way the unique characteristics of the OFC source with the flexibility of a scanning FP interferometer, we demonstrate a compact and versatile DFCS method with a resolution of ~20 MHz and a single-scan bandwith of ~1 THz, capable of absolute frequency measurements with a noise-equivalent-absorption level per comb mode of 2.7 · 10^−9 ^cm^−1^ Hz^−1/2^ in the near-infrared. This level can be further improved by increasing the optical path using an enhancement cavity instead of a multipass-cell (for example 10^−10 ^cm^−1^ Hz^−1/2^ could be readily obtained with a 1-km interaction length by an enhancement cavity finesse of ~3,000). In this case, an additional frequency-locking loop between the enhancement cavity and the optical frequency comb will have to be implemented increasing the overall complexity of the experimental configuration in the same way as for other cavity-enhanced spectroscopy setups. In addition, to further extend the optical bandwidth while maintaining the same measurement time and frequency resolution, the OTF may be replaced by a grating and a photodiode array (each pixel of the array analyzing a single micro-cavity *FSR*) placed at the end of the detection chain. The latter configuration would allow for a simultaneous detection of the intensity transmitted at different orders of the micro-cavity, greatly increasing the single-scan optical bandwidth. The detection scheme adopted is extremely compact, it is completely independent from the light source employed for the spectroscopy measurements and it does not require complicated servo-locking systems. Nevertheless its unique characteristics and performances allow one to measure molecular absorption profiles with extremely high precision. The detection scheme lends itself to be extended to other spectral regions ranging from UV to THz and can be adopted in a large variety of experiments from remote sensing to precision spectroscopy. Resolution and bandwidth are also further scalable by increasing the resonator finesse or by tuning the mirror distance. Finally, the compactness of the spectrometer makes it easily integrable in miniaturised systems, like lab-on-chip devices, especially in combination with micro-ring-based OFC comb sources.

## Methods

For the SMART DFCS validation experiments a commercial OFC (Menlo Systems, FC1500-250) has been used. The OFC is constituted by a dual-branch Er:fiber femtosecond oscillator operating with an average output power of 0.5 W in an optical bandwidth from 1500 to 1620 nm and a repetition frequency of 250 MHz a band from 1500 to 1620 nm. One output is coupled to a highly-nonlinear fiber for the one-octave supercontinuum generation from 1000 to 2100 nm, which feeds a home-made common path *f* − 2*f* interferometer detecting the comb offset frequency. The offset and repetition frequency of the comb are locked to a GPS-disciplined Rb frequency standard at 10 MHz (Precision Test Systems, GPS10RBN) by acting on the pump power and piezo cavity mirror of the low-power Er:fiber oscillator. The second amplified output is coupled into a single-mode fiber and combined with the output of a commercial fiber-coupled CW single-frequency laser (Agilent 81600B), tunable in the range from 1440 to 1650 nm with a 1-ms linewidth of 100 kHz. Both lasers are then filtered by a home-made tunable filter (optical bandwidth variable in the range 0.5 to 20 nm) and then sent through an aspherical fiber collimator to the FP micro-resonator. The fiber-coupled OTF has been realized by arranging the following optical components in a folded 4–*f* configuration: fiber-circulator (Thorlabs 6015-3-APC), diffraction-grating (Thorlabs GR25-0616), 75-mm focal-length AR-coated positive lens, variable slit, plane folding gold-mirror. The micro-resonator (Burleigh, RC-110) is constituted by two high-reflectivity (*T* = 0.005% at 1540 nm, Layertec) mirrors, a plane mirror and a spherical one with a radius of curvature of 1 m, spaced by ~150 *μ*m. A finesse of ~50,000 was measured in good agreement with mirror reflectivity specifications. A good mode-matching between the spatial mode of the incident beam with the fundamental TEM_00_ mode supported by the micro-cavity leads to a cavity throughput of ~56%, as measured for both the comb and CW reference laser, and leads to a spurious excitation of TEM_10_/TEM_01_ mode (5.3 GHz away from the main TEM_00_) and TEM_11_ (10.6 GHz away from the main TEM_00_) with a relative contrast of less than 1% and 0.1%, respectively. The FP microcavity transmitted beam is split into reference and probe beams. The probe beam is coupled to a Herriot-type multipass absorption cell (33-m interaction length) filled with C_2_H_2_ gas sample (99.5% purity). Two balanced photodetectors (Thorlabs, PDB410C) have been used to detect probe and reference beams. A passive 1.9 MHz low-pass RF-filter has been used to suppress high frequency noise components of the detector. The intensity scans have been recored by a 12-bit digital oscilloscope (Teledyne LeCroy HDO6034) with a sampling rate of 25 MS/s and processed by a 6-core OSX-based workstation (Mac Pro) equipped with MATLAB R2015b.

## Additional Information

**How to cite this article**: Gambetta, A. *et al*. Scanning micro-resonator direct-comb absolute spectroscopy. *Sci. Rep.* **6**, 35541; doi: 10.1038/srep35541 (2016).

## Figures and Tables

**Figure 1:**
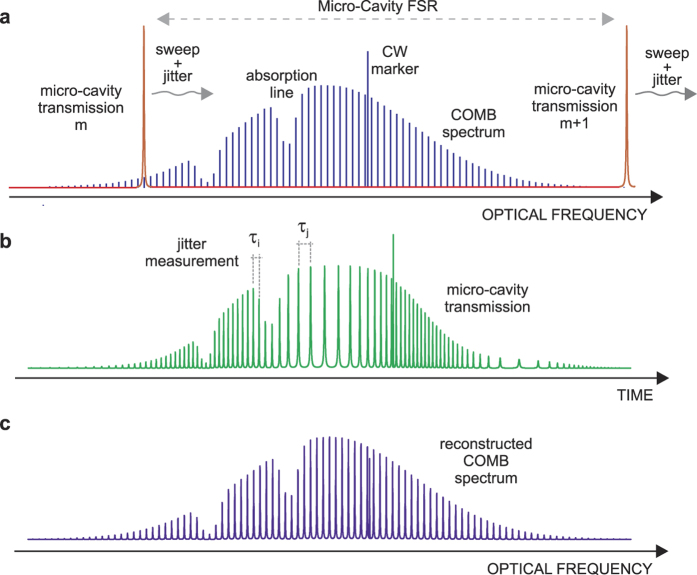
Operating principle of the SMART-DFCS method. (**a**) The comb optical spectrum (blu line) is sampled in time by the micro-cavity transmission resonance (red line). The cavity sweep is affected by environmental and electronic noise. (**b**) Micro-cavity transmission over scanning time. The comb line spacing in time (*τ*) is exploited to evaluate the jitter value along the measurement. (**c**) Optical spectrum of the comb as retrieved after jitter correction and absolute calibration.
